# The Na^+^/Ca^2+^ Exchanger 3 Is Functionally Coupled With the Na_V_1.6 Voltage-Gated Channel and Promotes an Endoplasmic Reticulum Ca^2+^ Refilling in a Transgenic Model of Alzheimer’s Disease

**DOI:** 10.3389/fphar.2021.775271

**Published:** 2021-12-08

**Authors:** Ilaria Piccialli, Roselia Ciccone, Agnese Secondo, Francesca Boscia, Valentina Tedeschi, Valeria de Rosa, Pasquale Cepparulo, Lucio Annunziato, Anna Pannaccione

**Affiliations:** ^1^ Division of Pharmacology, Department of Neuroscience, Reproductive and Dentistry Sciences, School of Medicine, Federico II University of Naples, Naples, Italy; ^2^ IRCSS SDN, Naples, Italy

**Keywords:** Na^+^/Ca^2+^ exchanger, NCX3, Na_V_1.6 channels, hippocampal neurons, Alzheimer’s disease, Tg2576 mice

## Abstract

The remodelling of neuronal ionic homeostasis by altered channels and transporters is a critical feature of the Alzheimer’s disease (AD) pathogenesis. Different reports converge on the concept that the Na^+^/Ca^2+^ exchanger (NCX), as one of the main regulators of Na^+^ and Ca^2+^ concentrations and signalling, could exert a neuroprotective role in AD. The activity of NCX has been found to be increased in AD brains, where it seemed to correlate with an increased neuronal survival. Moreover, the enhancement of the NCX3 currents (I_NCX_) in primary neurons treated with the neurotoxic amyloid *β* 1–42 (Aβ_1–42_) oligomers prevented the endoplasmic reticulum (ER) stress and neuronal death. The present study has been designed to investigate any possible modulation of the I_NCX_, the functional interaction between NCX and the Na_V_1.6 channel, and their impact on the Ca^2+^ homeostasis in a transgenic *in vitro* model of AD, the primary hippocampal neurons from the Tg2576 mouse, which overproduce the Aβ_1–42_ peptide. Electrophysiological studies, carried in the presence of siRNA and the isoform-selective NCX inhibitor KB-R7943, showed that the activity of a specific NCX isoform, NCX3, was upregulated in its reverse, Ca^2+^ influx mode of operation in the Tg2576 neurons. The enhanced NCX activity contributed, in turn, to increase the ER Ca^2+^ content, without affecting the cytosolic Ca^2+^ concentrations of the Tg2576 neurons. Interestingly, our experiments have also uncovered a functional coupling between NCX3 and the voltage-gated Na_V_1.6 channels. In particular, the increased Na_V_1.6 currents appeared to be responsible for the upregulation of the reverse mode of NCX3, since both TTX and the *Streptomyces griseolus* antibiotic anisomycin, by reducing the Na_V_1.6 currents, counteracted the increase of the I_NCX_ in the Tg2576 neurons. In agreement, our immunofluorescence analyses revealed that the NCX3/Na_V_1.6 co-expression was increased in the Tg2576 hippocampal neurons in comparison with the WT neurons. Collectively, these findings indicate that NCX3 might intervene in the Ca^2+^ remodelling occurring in the Tg2576 primary neurons thus emerging as a molecular target with a neuroprotective potential, and provide a new outcome of the Na_V_1.6 upregulation related to the modulation of the intracellular Ca^2+^ concentrations in AD neurons.

## Introduction

Alzheimer’s disease (AD) is one of the most common neurodegenerative disorders, with a clinical symptomatology ranging from cognitive disabilities to severe dementia (Querfurth and LaFerla, 2010). Dysfunctional ion channels and transporters have been implicated in neuronal loss and network disruption, thus emerging as a potential candidate responsible for neurodegeneration (Wada, 2006; Chakroborty and Stutzmann, 2014). Nonetheless, the remodelling of ionic homeostasis is historically considered a critical feature of the AD pathogenesis, being involved in neuronal and glial responses to amyloid β1-42 (Aβ_1–42_)-mediated injury (Berridge, 2010). However, despite the variety of studies aimed at exploring the role of ionic dyshomeostasis in the AD etiopathogenesis, including Ca^2+^ and Na^+^ dysregulation, many issues remain to be elucidated.

The involvement of the Na^+^/Ca^2+^ exchanger (NCX) in AD has been proposed in different studies (Colvin et al., 1991 and, 1994; Sokolow et al., 2011; Pannaccione et al., 2012; Pannaccione et al., 2020). Indeed, as a crucial regulator of intracellular Na^+^ and Ca^2+^ concentrations, NCX displays a neuroprotective role in many pathophysiological conditions affecting the central nervous system, thus emerging as a key target in neurodegeneration (Gomez-Villafuertes et al., 2007; Annunziato et al., 2020; Pannaccione et al., 2020). Although mainly considered as a Ca^2+^ extrusion mechanism, NCX works in fact in a bidirectional manner by mediating the Ca^2+^ influx along with the Na^+^ efflux (reverse mode) or, *vice versa*, the Ca^2+^ efflux and Na^+^ influx (forward mode) (Blaustein and Lederer, 1999). Of note, the proximity of NCX to different types of Na^+^ channels, including the voltage-gated sodium (Na_V_) channels, may render the exchanger an important source for Ca^2+^ influx (Poburko et al., 2007; Gershome et al., 2010).

Different reports have provided evidence about a neuroprotective role of NCX in the AD pathogenesis (Colvin et al., 1991 and, 1994; Pannaccione et al., 2012; Pannaccione et al., 2020). First, the increase of the NCX activity observed in surviving neurons in AD brain areas affected by neurodegeneration suggested that the exchanger could participate in the survival mechanisms occurring in AD neurons (Colvin et al., 1991). On the other hand, the modulation of the expression pattern of the three NCX isoforms, NCX1-3, was observed in AD brain tissues, with a marked loss of NCX3 in the parietal cortex of AD patients and in the synaptosomes from AD-affected brains (Sokolow et al., 2011). In agreement, we demonstrated that the upregulation of the NCX3 activity in primary hippocampal neurons exposed to Aβ_1–42_ oligomers was involved in neuronal survival in the early phase of Aβ_1–42_ injury (Pannaccione et al., 2012). In contrast, NCX3 dysfunction in the late phase of Aβ_1–42_ exposure determined neuronal death *via* endoplasmic reticulum (ER) stress and caspase-12 activation (Pannaccione et al., 2012). Intriguingly, a genome wide association study for the age at onset of AD identified SLC8A3, the gene encoding for NCX3, as a candidate gene for AD since its rare variants were shown to affect the age at onset of the disease (Saad et al., 2015).

Notably, we have recently reported that the expression and activity of the Na_V_1.6 channel subunit were upregulated in both primary hippocampal neurons exposed to exogenous Aβ_1–42_ oligomers and those from Tg2576 mice, a well-known transgenic model of AD (Ciccone et al., 2019). Na_V_1.6 channels, which are densely clustered at the axon initial segment (AIS) and at the nodes of Ranvier of myelinated axons, play a crucial role in the initiation and propagation of action potentials in excitable cells (Royeck et al., 2008; Akin et al., 2015; Solé and Tamkun, 2020). In line with several studies, our results showed that Na_V_1.6 channels were largely expressed not only at the AIS but also as somatic nanoclusters and that their expression was significantly increased in the soma and neurites of Tg2576 hippocampal neurons, hence determining their hyperexcitability (Akin et al., 2016; Sikora et al., 2017; Ciccone et al., 2019). Several studies have suggested that Na_V_ channels might collaborate with NCX to constitute a linkage between the Na^+^ and Ca^2+^ fluxes across the plasma membrane (Pappalardo et al., 2014; Radwański et al., 2016; Veeraraghavan et al., 2017; Torres, 2021). However, whether such cooperation may intervene in the neuronal Na^+^ and Ca^2+^ regulation in AD has not been explored yet.

Based on these considerations, the purpose of the present study has been to investigate by means of electrophysiological studies any possible changes in the NCX activity in primary hippocampal neurons from the Tg2576 mouse, a transgenic model overproducing the Aβ_1–42_ peptide. In addition, we have also assessed, through pharmacological and siRNA approaches, the possible involvement of a specific NCX isoform and explored the functional interaction between NCX and the Na_V_1.6 channel.

## Materials and Methods

### Animals

All the animals were handled according to the International Guidelines for Animal Research and the experimental protocols were approved by the Animal Care and Use Committee of the “Federico II” University of Naples. The heterozygous male Tg2576 mice and Wild Type (WT) females were purchased from a commercial source [B6; SJLTg(APPSWE)2576Kha, model 1349, Taconic, Hudson, NY].

### PCR Analysis

The genomic DNA from embryonic brain tissues was isolated by salt precipitation. Briefly, embryonic brain tissues were harvested during cerebral dissection and then thawed and homogenized with the TRI-reagent (SigmaAldrich, Milan, Italy). After adding one volume of chloroform to each sample, the DNA was precipitated with 100% ethanol and centrifuged at 4°C for 5 min at 16,000 × g. The DNA pellet was dried at room temperature and then re-suspended in Tris-EDTA buffer. The following primers were used to amplify the DNA region with the human APP Swedish mutation on both types of genomic DNA: 5′-CTG​ACC​ACT​CGA​CCA​GGT​TCT​GGG​T-3′ and 5′GTG​GAT​AAC​CCC​TCC​CCC AGCCTAGACCA-3′ (Eurofins Genomics, Ebersberg, Germany). The DNA was amplified as previously described (Ciccone et al., 2019) to detect the transgenic genotype.

### Primary Hippocampal Neurons

Primary neuronal cultures were prepared from the hippocampi of embryonic day 15 WT and Tg2576 mise as described by Ciccone et al. (2019). The cells were plated on 35 mm culture dishes coated with poly(d)-lysine hydrobromide Molecular Weight >300,000 (Sigma Aldrich, Milan, Italy), or onto 25 mm glass coverslips (Glaswarenfabrik Karl Hecht KG, Sondheim, Germany), coated with 100 μg/ml poly(d)-lysine hydrobromide Molecular Weight 30,000–70,000 (Sigma Aldrich, Milan, Italy), at a density of one embryo hippocampus/1 ml 10 μM of cytosine β-d-arabinofuranoside (Sigma Aldrich, Milan, Italy) were added 3 days after plating to inhibit non-neuronal cell growth. The neurons were cultured at 37°C in a humidified 5% CO_2_ atmosphere. The experiments were performed not earlier than 8 days *in vitro* (DIV).

### Electrophysiological Recordings: NCX and Na^+^ Currents

The NCX and Na^+^ currents (I_NCX_; I_Na_, respectively) were recorded in primary hippocampal neurons from the Tg2576 and WT mice by means of the patch-clamp technique in a whole-cell configuration using the commercially available amplifier Axopatch 200B and the Digidata 1322 A interface (Molecular Devices) as previously described (Molinaro et al., 2008; Pannccione et al., 2012; Secondo et al., 2015; Ciccone et al., 2019). The data were acquired and analyzed using the pClamp software (version 9.0, Molecular Devices). The I_NCX_ were recorded starting from a −60 mV holding potential up to a short-step depolarization at +60 mV as previously described (Molinaro et al., 2008; Pannccione et al., 2012). In particular, reverse mode of NCX is represented in rising portion of the ramp ranging from 0 mV to +60 mV and measured at the end of +60 mV whereas Forward mode is represented in the descending portion of the ramp (from −120 to −0 mV) and measured at the end of −120 mV.The I_NCX_, sensitive to Ni^2+^, were isolated by subtracting the Ni^2+^-insensitive components from the total currents (I_NCX_ = I_TOT_-I_Ni-Resistent_). The tetrodotoxin (TTX)-sensitive I_Na_ were recorded using low resistance electrodes (1.4–2.3 MΩ), sampled at a rate of 100 kHz and filtered at 5 kHz. The neurons were held at −120 mV and stepped to a range of potentials (−100 to +30 mV in 10 mV increments) as reported previously (Secondo et al., 2015; Ciccone et al., 2019). The neurons were perfused with external Ringer’s solution containing the following (in mM): 126 NaCl, 1.2 NaHPO_4_, 2.4 KCl, 2.4 CaCl_2_, 1.2 MgCl_2_, 10 glucose, and 18 NaHCO_3_, pH7.4. Tetraethylammonium (TEA) and nimodipine (20 mM and 10 μM, respectively) were added to the external solution in order to block the potassium and calcium currents. The pipettes were filled with 100 K-gluconate, 10 TEA, 20 NaCl, 1 Mg-ATP, 0.1 CaCl_2_, 2 MgCl_2_, 0.75 EGTA, and 10 HEPES, adjusted to pH 7.2 with Cs(OH)_2_. Any possible changes in cell size were calculated by monitoring the capacitance of each cell membrane, which is directly related to the membrane surface area, and by expressing the current amplitude data as current densities (pA/pF). The capacitive currents were estimated from the decay of the capacitive transient induced by 5 mV depolarizing pulses from a holding potential of −80 mV and acquired at a sampling rate of 50 kHz. The capacitance of the membrane was calculated according to the following equation: Cm = τc•Io/ΔEm(1-I∞/Io), where Cm is the membrane capacitance, τc is the time constant of the membrane capacitance, Io is the maximum capacitance current value, ΔEm is the amplitude of the voltage step, and I∞ is the amplitude of the steady-state current (Pannaccione et al., 2012).

### [Ca^2+^]_i_ Measurement

Hippocampal neurons were incubated with 10 µM Fura-2 AM for 30 min at 37°C in normal Krebs solution containing 5.5 mM KCl, 160 mM NaCl, 1.2 mM MgCl_2_, 1.5 mM CaCl_2_, 10 mM glucose, and 10 mM HEPES-NaOH (pH 7.4). At the end of the loading period, coverslips were placed into a perfusion chamber (Medical System Co., Greenvale, NY, United States), mounted onto the stage of an inverted Zeiss Axiovert 200 microscope (Carl Zeiss, Milan, Italy), equipped with a FLUAR 40X oil objective lens. The experiments were carried out with a digital imaging system composed of a MicroMax 512BFT cooled CCD camera (Princeton Instruments), LAMBDA10-2 filter wheeler (Sutter Instruments), and Meta-Morph/MetaFluor Imaging System software (Universal Imaging). Primary neurons were alternatively illuminated at wavelengths of 340 and 380 nm by a Xenon lamp. The emitted light was passed through a 512 nm barrier filter. Fura-2 fluorescence intensity was measured every 3 s. Fura-2 ratiometric values were automatically converted by MetaMorph/MetaFluor Imaging System software (Universal Imaging) to cytosolic Ca^2+^ levels, by using a preloaded calibration curve obtained in preliminary experiments, as previously described (Grynkiewicz et al., 1985).

To elicit ER Ca^2+^ release in neurons, ATP (100 µM) and the irreversible inhibitor of sarco-endoplasmic reticulum Ca^2+^ ATPase (SERCA) pump thapsigargin (1 µM) were added in a Ca^2+^-free solution (containing 5.5 mM KCl, 160 mM NaCl, 1.2 mM MgCl_2_, 10 mM glucose, and 10 mM HEPES-NaOH, pH 7.4), as indicated by the bar of Figure 3. Specifically, ATP was able to trigger a rapid ER Ca^2+^ release by activating its plasmalemmal purinergic receptors coupled to Gq, while thapsigargin, by blocking SERCA, inhibits tonic refilling into ER and determines a progressive and slow ER Ca^2+^ release. The use of both tools allows to recruit all components of ER Ca^2+^ store (Caputo et al., 2012; Criscuolo et al., 2019; Secondo et al., 2019; Tedeschi et al., 2019; Tedeschi et al., 2021).

### Western Blotting

Total lysates for the immunoblotting analyses were obtained as follows: the primary hippocampal neurons were washed in phosphate buffered saline (PBS) and collected by gentle scraping in ice-cold RIPA buffer containing (in mM) 50 Tris pH 7.4, 100 NaCl, 1 EGTA, 1 PMSF, 1 sodium orthovanadate, 1 NaF, 0.5% NP-40, and 0.2% SDS supplemented with protease inhibitor cocktail II (Roche Diagnostic, Monza, Italy). The nitrocellulose membranes were incubated with the following antibodies: rabbit-polyclonal anti-NCX3, anti-NCX1, anti-NCX2 (1:1,000, Alomone Labs, Israel) and anti-β-actin peroxidase (1:10,000, Sigma-Aldrich, Milan, Italy). The immunoreactive bands were detected with thechemiluminescence system (Amersham-Pharmacia-Biosciences, UK). The films were developed with a standard photographic procedure and the quantitative analysis of the bands detected was carried out by densitometric scanning.

### Confocal Immunofluorescence Analysis

The confocal immunofluorescence procedures in neuronal cultures were performed as previously described (Boscia et al., 2017; de Rosa et al., 2019). The cell cultures were fixed in 4% wt/vol paraformaldehyde in phosphate buffer for 30 min. After blocking with 3% BSA, the cells were incubated with monoclonal anti-NCX3 (1:1,000, Trans Genic Inc., Japan) and rabbit policlonal anti-Na_V_1.6 (1:1,000, Alomone Labs, Israel). Next, the cells were incubated with Alexa594-conjugated anti-mouse IgGs and biotinylated anti-rabbit antibodies. NCX3 was detected by using the tyramide signal amplification (TSA) fluorescein system (Perkin-Elmer, Life Sciences). Hoechst 33258 was used to stain the nuclei. The images were observed using a Zeiss LSM 700 laser (Carl Zeiss) scanning confocal microscope. The single images were taken with an optical thickness of 0.7 μm and a resolution of 1024 × 1024. The NCX3 and Na_V_1.6 fluorescence intensities were quantified in terms of pixel intensity by using the NIH image software, as previously described (Boscia et al., 2012). Briefly, digital images were taken with 63× objective and identical laser power settings and exposure times were applied to all the photographs from each experimental set. The co-localization between NCX3 and Na_V_1.6 was analysed by line profiling the Cy3 (red) and FITC (green) fluorescence intensities using the ZEN lite software (Carl Zeiss) (Cammarota et al.,2021).

### Statistical Analysis

GraphPad Prism 6.02 was used for the statistical analyses (GraphPad Software, La Jolla, CA). Thedata are expressed as the mean ± S.E.M. of the values obtained from individual experiments. The statistical comparisons between the groups were performed by means of the Student’s t-test or one-way analysis of variance (ANOVA) followed by the Bonferroni post hoc test or Newman–Keuls’ test. *p* < 0.05 was considered significant.

## Results

### The Activity of NCX Is Significantly Upregulated in the Reverse Mode of Operation in the Tg2576 Hippocampal Neurons

**FIGURE 1:**
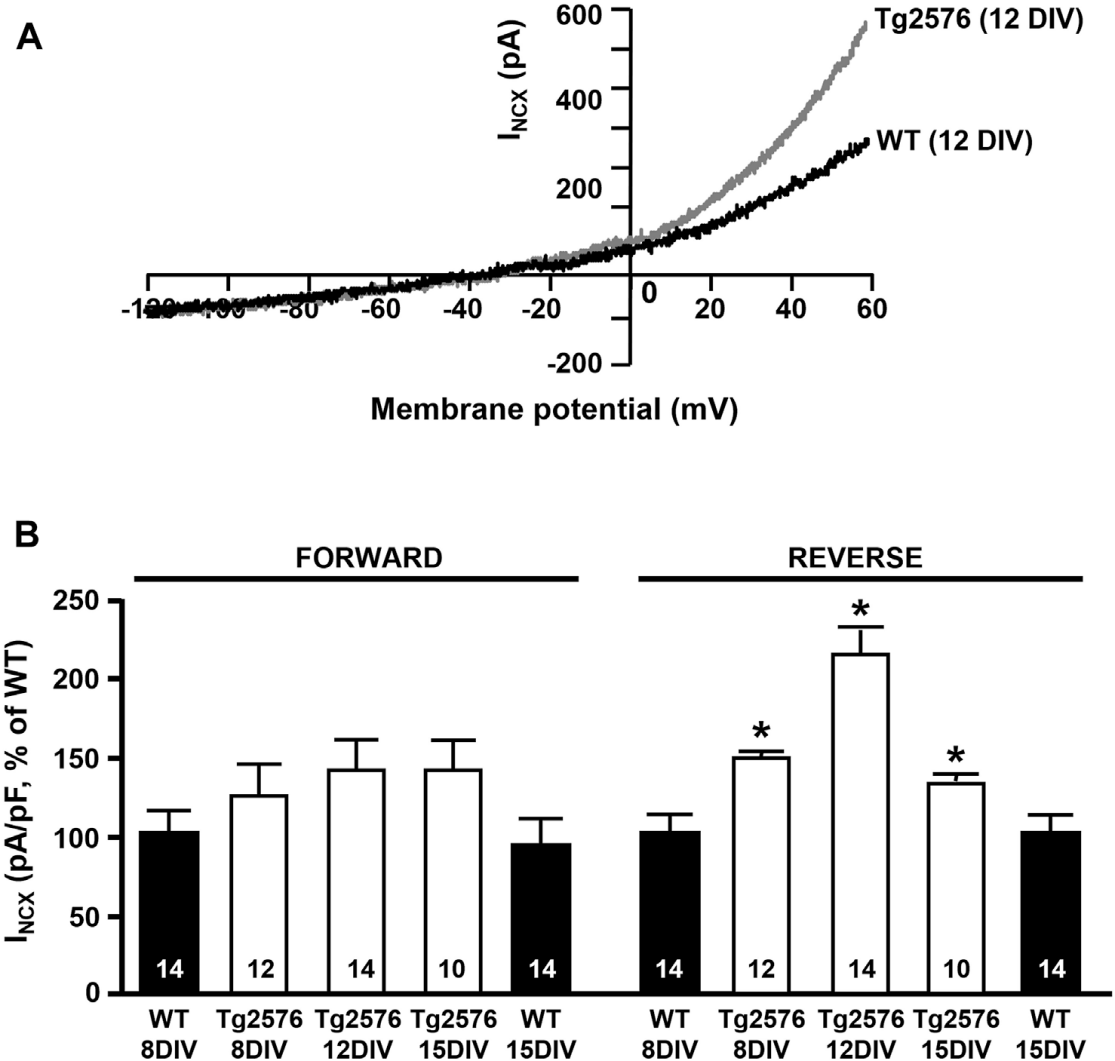
I_NCX_ in WT and Tg2576 primary hippocampal neurons. **(A)** Representative superimposed traces of I_NCX_ in the reverse and forward modes of operation recorded in WT (black trace) and Tg2576 (gray trace) primary hippocampal neurons at 12 DIV. **(B)** Quantification of I_NCX_ in the reverse and forward modes of operation recorded in WT and Tg2576 primary hippocampal neurons at 8, 12, and 15 DIV, expressed as percentage of increase in comparison to WT. Values are expressed as mean ± SEM of 3 independent experimental sessions. Statistical comparisons between groups were performed by one-way ANOVA followed by Newman-Keuls’ test. (*p < 0.05 vs. WT). The number of cells used for each experimental condition is noted on the bars.

First, we examined any possible changes of the NCX activity in the Tg2576 hippocampal neurons. We assessed the I_NCX_ in the forward and reverse modes of operation by patch-clamp experiments in a whole-cell configuration in both the WT and Tg2576 cultured hippocampal neurons after 8, 12, and 15 DIV. Electrophysiological recordings showed that the NCX activity was significantly modulated in a time-dependent manner only in the reverse mode of operation in the Tg2576 hippocampal neurons, while the forward mode was not affected (Figures 1A,B). In particular, the I_NCX_ in theTg2576 neurons were increased at 8, 12, and 15 DIV in comparison with those recorded in the WT neurons at the same DIV, with a marked peak at 12 DIV (Figure 1A,B).

### NCX3 Silencing or Pharmacological Inhibition Prevents the Upregulation of the I_NCX_ in the Tg2576 Hippocampal Neurons

**FIGURE 2:**
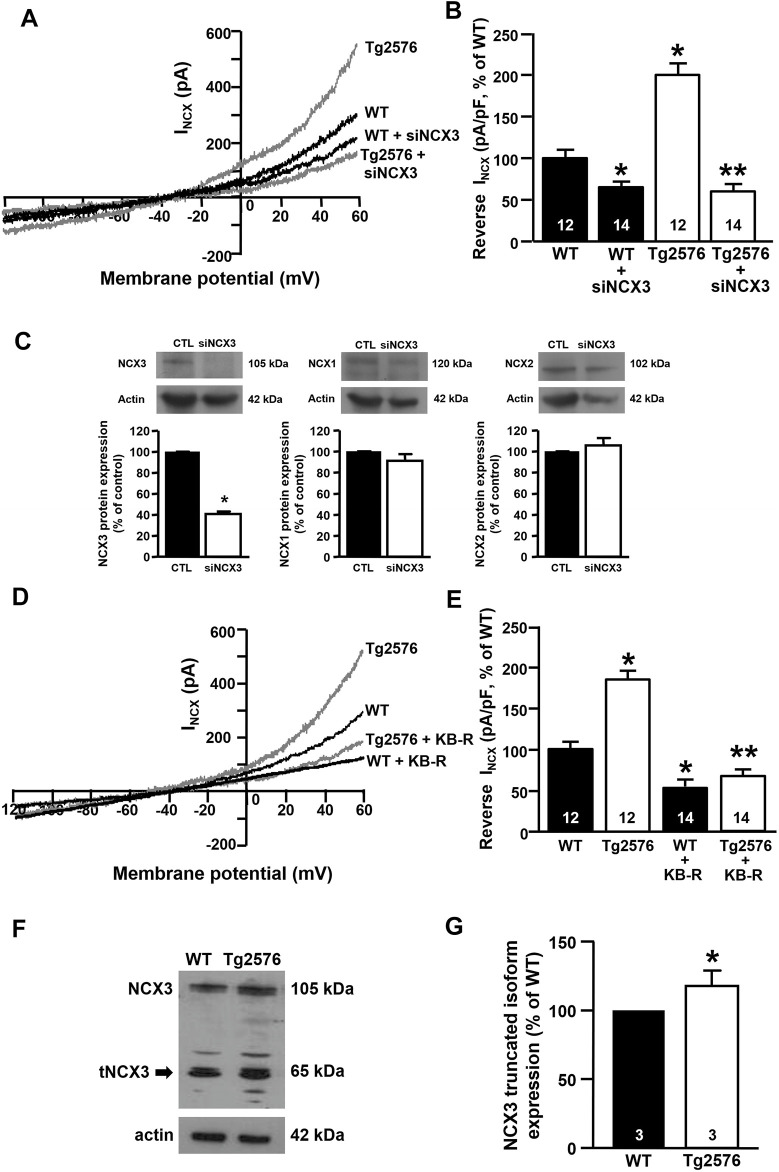
Effect of NCX3 silencing or inhibition by KB-R7943 in WT and Tg2576 primary hippocampal neurons. **(A)** Representative superimposed traces of I_NCX_ in the reverse and forward modes of operation recorded in WT and WT plus siNCX3 (black traces), Tg2576 and Tg2576 plus siNCX3 (grey traces) primary hippocampal neurons at 12 DIV. **(B)** Quantification of I_NCX_ in the reverse mode of operation represented in A, expressed as percentage of variation in comparison to WT. Values are expressed as mean ± SEM of 3 independent experimental sessions. **(C)** Representative Western blotting experiments and relative quantifications showing the effect of NCX3 silencing (siNCX3) on NCX3, NCX1, and NCX2 protein expression in primary hippocampal neurons **(D)** Representative superimposed traces of I_NCX_ in the reverse and forward modes of operation recorded from WT and WT plus 0.5 μM kB-R7943 (black traces), Tg2576 and Tg2576 plus 0.5 μM kB-R7943 (grey traces) primary hippocampal neurons at 12 DIV. **(E)** Quantification of I_NCX_ in the reverse mode of operation represented in D, expressed as percentage of variation in comparison to WT. Values are expressed as mean ± SEM of 3 independent experimental sessions. The number of cells used for each experimental condition is noted on the bars. **(F, G)** Representative Western blot of NCX3 protein expression and densitometric quantification of NCX3 truncated band in WT and Tg2576 primary hippocampal neurons at 12 DIV, represented as percentage of WT. Values are expressed as mean ± SEM of 3 independent experimental sessions. Statistical comparisons between groups were performed by one-way ANOVA followed by Newman-Keuls’ test. (*p < 0.05 vs. WT; **p < 0.05 vs. Tg2576 mice).

Patch-clamp experiments revealed that the silencing of NCX3 (siNCX3) prevented the upregulation of the I_NCX_ in the reverse mode in the Tg2576 hippocampal neurons (Figures 2A,B). Of note, the siRNA directed against NCX3 did not modify the expression of the other two NCX isoforms, NCX1 and NCX2 (Figure 2C). Moreover, the specific contribution of NCX3 to the I_NCX_ upregulation in the reverse mode was further confirmed by blocking the exchanger with the 2-{2-[4-(4-nitrobenzyloxy) phenyl]ethyl}isothiourea mesylate (KB-R7943). Of note, KB-R7943 was demonstrated to be three-fold more inhibitory to NCX3 than to NCX1 and NCX2, with IC_50_ values of 4.9 ± 0.4 μM and 4.1 ± 0.3 μM for NCX1 and NCX2, respectively, and of 1.5 ± 0.1 μM for NCX3 (Iwamoto and Shigekawa, 1998). Additionally, KB-R7943 at low concentrations inhibits the NCX3 activity preferentially in the reverse mode (IC_50_ = 1.1 ± 3.4 μM for the reverse mode and IC_50_ > 30 μM for the forward mode) (Iwamoto and Shigekawa, 1998; Watano et al., 1996; Annunziato et al., 2004). In particular, preliminary concentration/response experiments revealed that 0.5 μM kB-R7943 was the minimum concentration able to significantly inhibit NCX3 without interfering with NCX1 and NCX2 isoforms. Importantly, electrophysiological recordings showed that in the presence of KB-R7943 at the concentration of 0.5 μM, the upregulation of the I_NCX_ in the reverse mode in the Tg2576 hippocampal neurons was completely prevented, with the return of the I_NCX_ to levels similar to those of the WT neurons (Figures 2D,E). Interestingly, as previously observed in hippocampal neurons exposed to Aβ_1–42_ oligomers (Pannaccione et al., 2012), Western blot analyses revealed that the Tg2576 neurons displayed an upregulation of the NCX3 truncated band migrating at around 65 kDa in comparison with the WT neurons (Figures 2F,G).

### The Enhancement of the NCX3 Activity in the Reverse Mode Participates to the Filling State of ER but Not to the Maintenance of Cytosolic Ca^2+^ Levels in the Tg2576 Hippocampal Neurons

**FIGURE 3:**
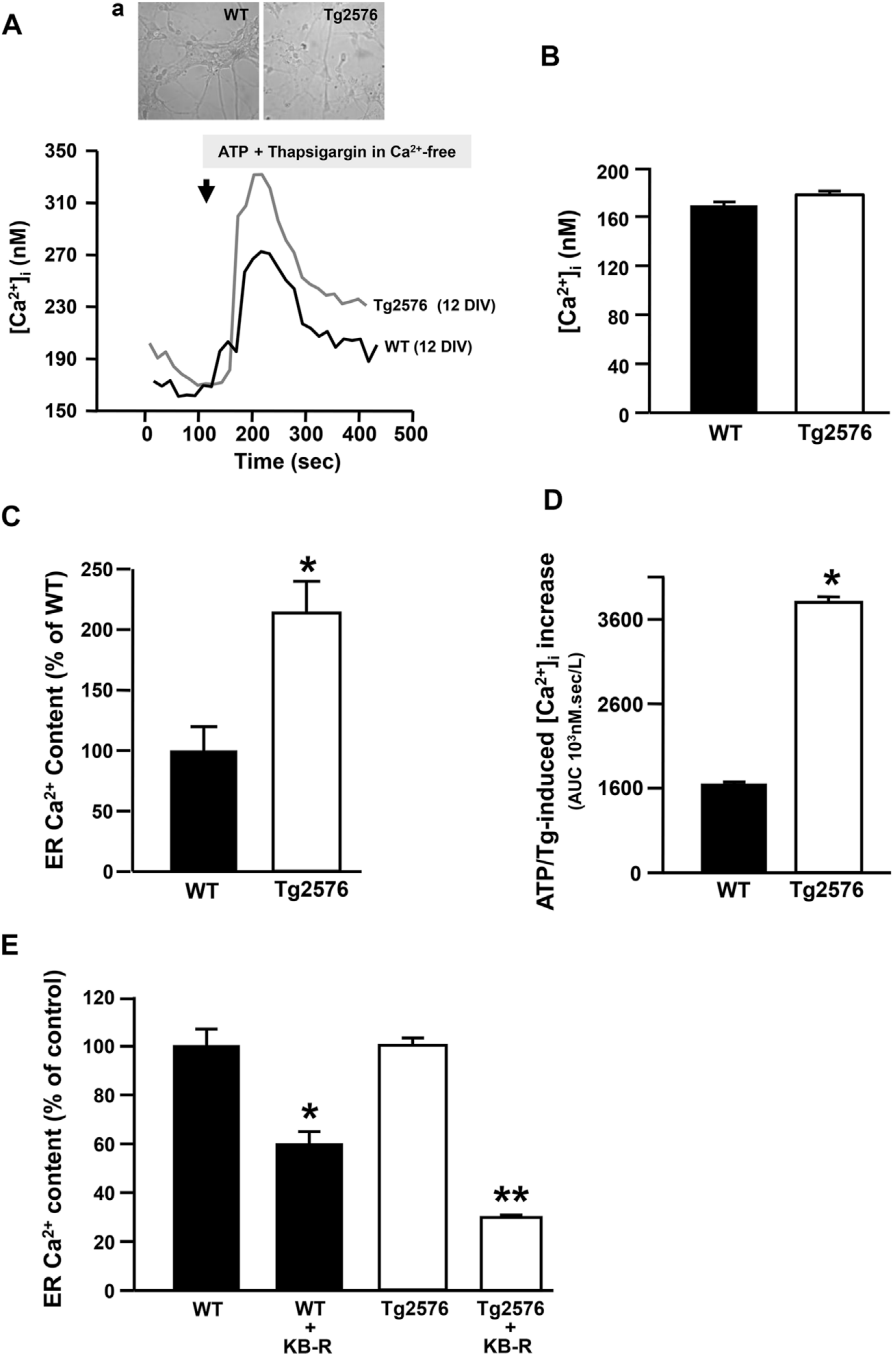
Effect of NCX3 inhibition by KB-R7943 on ER Ca^2+^ content in WT and Tg2576 primary hippocampal neurons. **(A)** Representative superimposed traces of [Ca^2+^]_i_ measured in WT (black trace, *N* = 36) and Tg2576 (grey trace, *N* = 34) primary hippocampal neurons at 12 DIV, representative images in panel **(A)**. **(B)** Quantification of basal values of [Ca^2+^]_i_ in WT (*N* = 36) and Tg2576 primary hippocampal neurons at 12 DIV (*N* = 34). **(C)** ER Ca^2+^ content quantified as [Ca^2+^]_i_ increase induced by Thapsigargin (Tg; 1 μM) and ATP (100 μM) in 0 μM Ca^2+^, and expressed as percentage of the effect observed in WT (considered as 100%). **(D)** AUCs of [Ca^2+^]_i_ calculated for **(A)**. **(E)** Quantification of ER Ca^2+^ content in WT and Tg2576 primary hippocampal neurons at 12 DIV treated with KB-R7943 at 0.5 μM. Values are represented as percentage of respective controls, expressed as mean ± SEM of 3 independent experimental sessions. Statistical comparisons between groups were performed by one-way ANOVA followed by Newman-Keuls’ test. (*p < 0.05 vs. WT; **p < 0.05 vs. Tg2576 mice).

To study the intracellular Ca^2+^ homeostasis and the putative relationship between NCX3 and the ER Ca^2+^ content in the Tg2576 hippocampal neurons, we performed Ca^2+^ imaging analyses with the fluorescent Ca^2+^ indicator Fura-2AM. The [Ca^2+^]_i_ in the Tg2576 hippocampal neurons did not differ from that measured in the WT neurons (Figures 3A,B) unlike the ER Ca^2+^ levels (Figures 3A,C). Indeed, the exposure to ATP plus the SERCA inhibitor thapsigargin, both triggering an ER Ca^2+^ release, determined a significantly higher increase in [Ca^2+^]_i_ and in the area under the curve (AUC) value in the Tg2576 hippocampal neurons compared with those in the WT neurons (Figures 3A,D).

In particular, we measured a significant difference between WT and Tg2576 when ATP plus thapsigargin was added to 0 mM extracellular Ca^2+^, showing that the simultaneous activation of NCX reverse mode is not necessary to measure changes in ER Ca^2+^ levels. This suggested that the addition of ATP and thapsigargin may unmask a tonic and stable ER Ca^2+^ dysfunction in Tg2576 neurons. Moreover, to study the putative contribution of NCX3 isoform to the filling state of ER Ca^2+^ stores, basal and ER Ca^2+^ levels were measured in the absence or presence of the well known NCX inhibitor KB-R7943. Specifically, KB-R7943 was preincubated for 20 min in a Ca^2+^-containing solution at the final concentration of 0.5 μM, selectively inhibiting the NCX3 reverse activity. Under these conditions, it significantly reduced the amount of Ca^2+^ released from the ER both in the WT hippocampal neurons and in the Tg2576 neurons (Figure 3E). However, this effect was significantly greater in the Tg2576 neurons than in the WT neurons (Figure 3E).

### The Functional Coupling Between Na_V_1.6 Channels and NCX3 Exchangers Underlies the Increased Reverse Activity of NCX3 in theTg2576 Hippocampal Neurons

**FIGURE 4:**
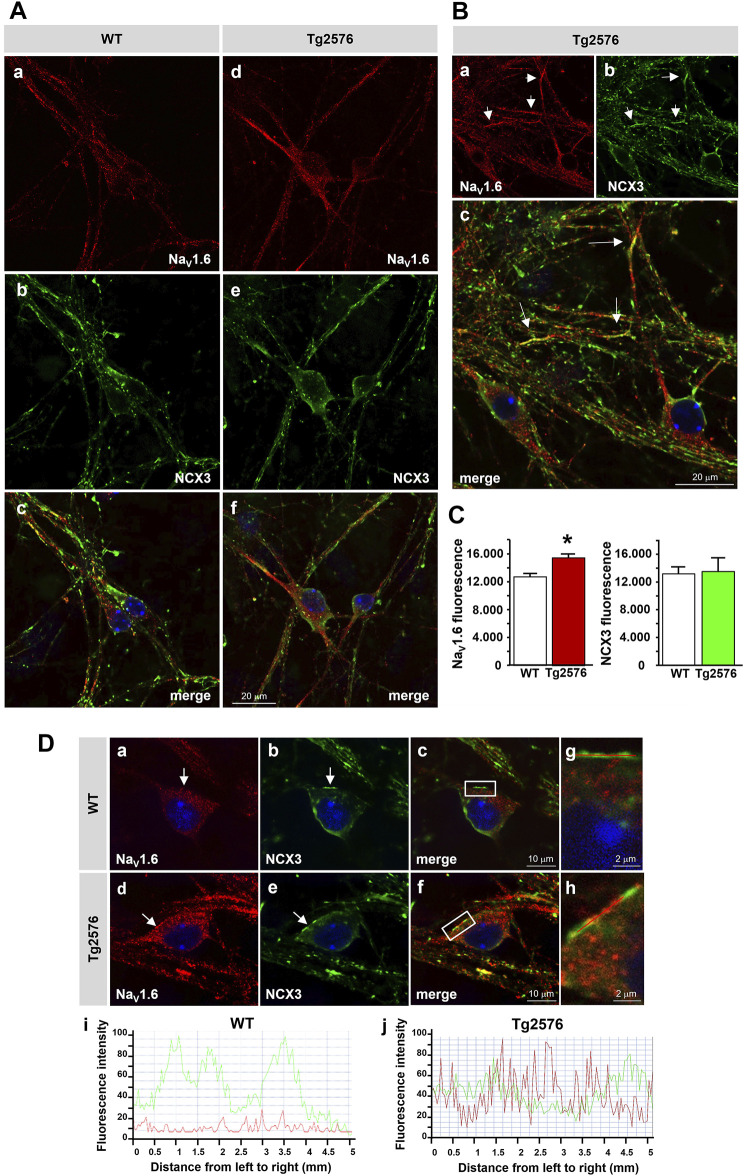
Distribution of Na_V_1.6 channels and NCX3 in Tg2576 primary hippocampal neurons. **(A)** Confocal microscopic images displaying the distribution of Na_V_1.6 (red) and NCX3 (green) immunoreactivities in hippocampal neurons isolated from WT and Tg2576 mouse embryos and cultured for 12 DIV (scale bars: in a–f: 20 µm). **(B)** Confocal microscopic images displaying the distribution of Na_V_1.6 (red) and NCX3 (green) immunoreactivities in Tg2576 primary hippocampal neurons at 12 DIV. Arrows in a-c point to the intense co-localization of Na_V_1.6 and NCX3 immunostaining along neurites (scale bars: 20 µm). **(C)** Densitometric analysis of Na_V_1.6 **(left)** and NCX3 **(right)** fluorescence intensities in WT and Tg2576 neurons at 12 DIV. The data are expressed in arbitrary units (*p < 0.05 vs. WT). **(D)** Confocal microscopic images displaying the distribution of Na_V_1.6 (red) and NCX3 (green) immunoreactivities in WT and Tg2576 primary hippocampal neurons at 12 DIV. Arrows in a–f point to Na_V_1.6 and NCX3 immunoreactivities along the somatic plasma membrane of both WT and Tg2576 neurons. Panels g and h show higher magnification images of the frame depicted in c and f, respectively. Nuclei were counterstained with DAPI (blue) (scale bars: in a–f: 10 μm; in g and h: 2 µm). Panels i and j show the line profiling of Na_V_1.6 (red) and NCX3 (green) fluorescence intensities along the line selected on the somatic plasma membrane of both WT (c) and Tg2576 (f) hippocampal neurons.

We previously showed that the expression and activity of Na_V_1.6 channels were time-dependently upregulated in the Tg2576 hippocampal neurons, with a maximum increase at 12 DIV (Ciccone et al., 2019). To explore whether the neuronal NCX3 and Na_V_1.6 functions might be coupled, we first investigated the co-expression of NCX3 and Na_V_1.6 in the 12 DIV Tg2576 hippocampal neurons. In line with our previous observations (Ciccone et al., 2019), quantitative immunofluorescence analyses showed that the Na_V_1.6 immunofluorescence significantly increased intracellularly and along the plasma membrane of both the soma and neurites of the 12 DIV Tg2576 hippocampal neurons compared with the WT cultures (Figures 4A–C). Although the global immunofluorescence signal of NCX3 remained unaltered in the Tg2576 neurons compared to the WT, a clustered co-expression of the upregulated Na_V_1.6 channels with NCX3 was clearly detected along several plasma membrane and cytosolic domains of both the soma and neurites of the Tg2576 neurons (Figures 4A–C).

**FIGURE 5:**
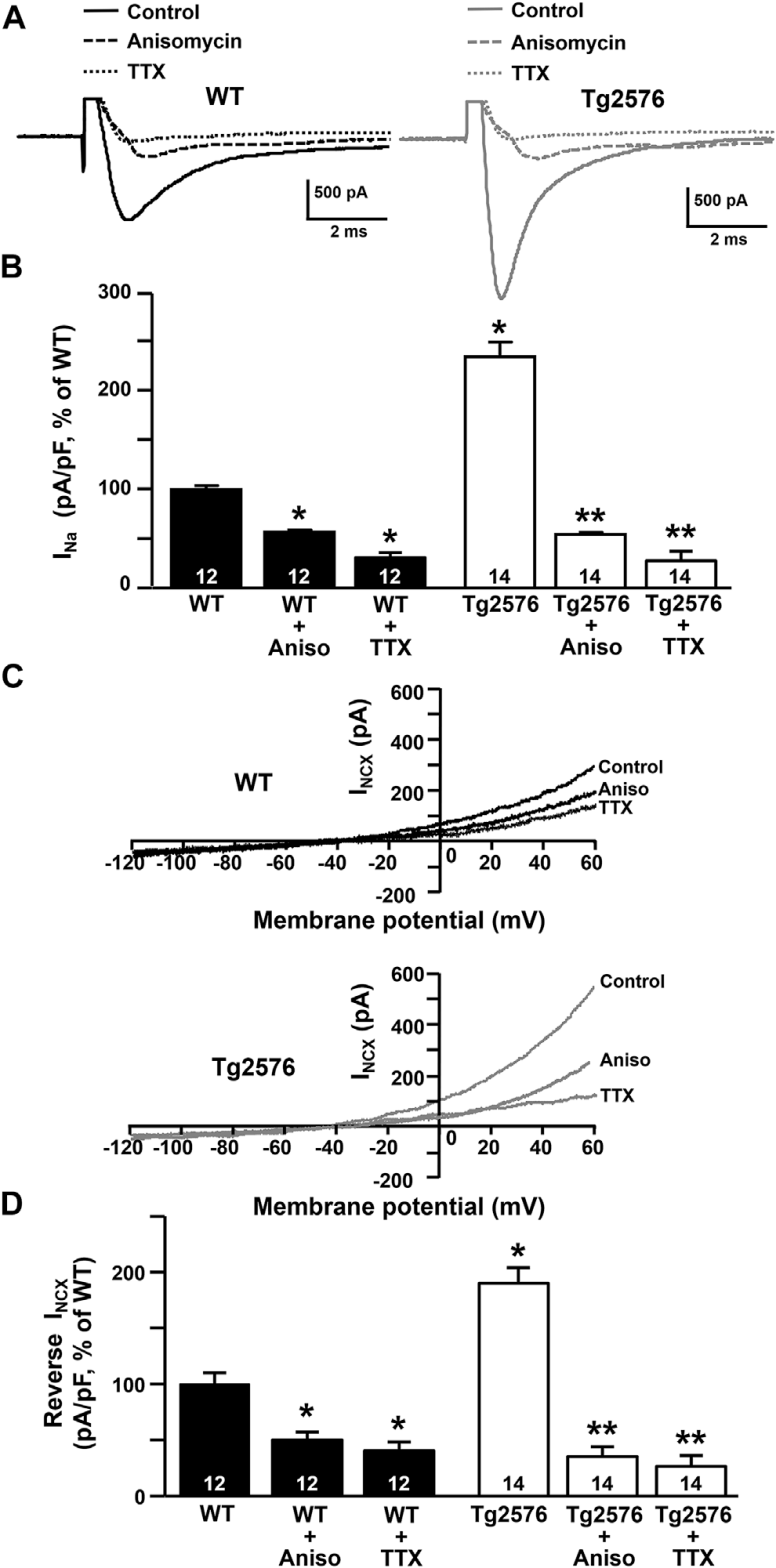
Effect of TTX and anisomycin on I_Na_ and I_NCX_ in WT and Tg2576 primary hippocampal neurons. **(A)** Representative traces of I_Na_ recorded in control conditions and in the presence of TTX or anisomycin pre-treatment in WT (black traces) and Tg2576 (grey traces) primary hippocampal neurons at 12 DIV. **(B)** Quantification of I_Na_ represented in A, expressed as percentage of WT in control conditions. Values are expressed as mean ± SEM of 3 independent experimental sessions. **(C)** Representative superimposed traces of I_NCX_ in the reverse and forward modes of operation recorded in control conditions and in the presence of TTX or anisomycin pre-treatment in WT **(top)** and Tg2576 **(bottom)** primary hippocampal neurons at 12 DIV. **(D)** Quantification of I_NCX_ in the reverse mode of operation represented in C, expressed as percentage of WT in control conditions. Values are expressed as mean ± SEM of 3 independent experimental sessions. Statistical comparisons between groups were performed by one-way ANOVA followed by Newman-Keuls’ test (*p < 0.05 vs. WT; **p < 0.05 vs. Tg2576 mice). The number of cells used for each experimental condition is noted on the bars.

Next, to test the hypothesis that the reversal of the NCX3 activity was driven by the increased Na^+^ inward currents mediated by the Na_V_1.6 channels, we measured both the I_Na_ and I_NCX_ in the presence of the sodium channel blocker TTX added to the extracellular recording solution, or after the treatment with anisomycin, a *Streptomyces griseolus* antibiotic that, by promoting p38 mitogen-activated protein (MAP) kinase activation, induces the selective endocytosis of Na_V_1.6 and, subsequently, the reduction of the Na_V_1.6-mediated currents (Wittmack et al., 2005; Gasser et al., 2010; Ciccone et al., 2019). We observed that the I_Na_ recorded in the presence of TTX or after the treatment with anisomycin were significantly reduced in both the WT and Tg2576 hippocampal neurons (Figures 5A,B). Moreover, both the pharmacological tools, TTX and anisomycin, were able to counteract the increase of the I_NCX_ in the reverse mode of operation not only in the Tg2576 hippocampal neurons but also in the WT neurons, despite the extent of the I_NCX_ reduction was greater in the Tg2576 neurons than in the WT neurons (Figures 5C,D).

## Discussion

The impact of the modulation of NCX on neuronal survival in AD has been investigated in several studies. Notably, a recent genome-wide association study by Saad and co-workers identified *SLC8A3*, the gene encoding for NCX3, among different genes in which multiple rare variations were associated with the age of onset of AD, thus proposing the exchanger as a possible molecular factor determining the timing of the onset of the disorder (Saad et al., 2015). However, although different reports have suggested a neuroprotective role of NCX in different experimental models of AD, further efforts should be made to characterize the involvement of the exchanger in the onset of AD. Many studies have demonstrated that the failure of the machinery regulating Ca^2+^ homeostasis is a crucial event in the AD etiopathogenesis (Berridge, 2010). Of note, disturbances in Ca^2+^ homeostasis were demonstrated to occur before the development of overt AD symptoms (Etcheberrigaray et al., 1998). This evidence suggested that the alteration of the systems regulating [Ca^2+^]_i_ may be an upstream event in the AD pathogenesis, inducing the early changes in learning and memory functions. In the present study, we have therefore moved to assess any possible modulation of the NCX activity and its impact on the [Ca^2+^]_i_ in primary hippocampal neurons from the Tg2576 mouse, an *in vitro* model of AD. Primary cultures from the Tg2576 mouse, which carries the APP_SWE_ double mutation of the amyloid precursor protein, accumulate Aβ_1–42_ over time in culture, thus recapitulating some of the main features of the Aβ-induced neurodegeneration (Takahashi et al., 2004; Almeida et al., 2005; Takahashi et al., 2013).

Interestingly, we observed that the I_NCX_ were increased in the reverse, Ca^2+^ influx mode, while the forward mode was not affected. The enhancement of the I_NCX_ appeared to be mediated by a specific NCX isoform, NCX3, since both the silencing of NCX3 and the treatment with the isoform-selective inhibitor KB-R7943 significantly reduced the reverse I_NCX_ in the Tg2576 neurons. Interestingly, the upregulation of the NCX3 currents in the Tg2576 hippocampal neurons was time-dependent, with a maximum increase at 12 DIV. In agreement, a previous *in vitro* study by our group had demonstrated that NCX3 was modulated after the exposure of primary hippocampal neurons to synthetic Aβ_1–42_ oligomers, thus strongly implicating NCX3 in the neuronal responses to Aβ_1–42_ injury (Pannaccione et al., 2012). Of note, the upregulation of the activity of NCX3 was associated with an increased formation of its truncated isoform, which was demonstrated to be hyperfunctional (Pannaccione et al., 2012). In line with this finding, we observed that the maximum increase of the activity of NCX3 was concomitant with the over-expression of its truncated isoform also in the Tg2576 hippocampal neurons, as Western blot analyses revealed a marked increase of the NCX3 band migrating at around 65 kDa in the 12 DIV Tg2576 neuronal lysates in comparison with the WT lysates. Interestingly, different truncated isoforms of NCX3, as well as of NCX1, corresponding to any splicing variants or cleavage products, have been found in the brain by different research groups and have been demonstrated to be functional or even hyper-functional, probably due to the loss of regulatory domains (Gabellini et al., 1996; Van Eylen et al., 2001; Lindgren et al., 2005; Michel et al., 2015; Michel et al., 2016). Of note, our group, and others, have shown that the cleavage of NCX3 by calpains could be a form of post-translational regulation providing a hyperactive NCX3 isoform by increasing, in particular, the reverse mode capacity of the exchanger (Pannaccione et al., 2012; Michel et al., 2016; Cammarota et al., 2021).

Ca^2+^ entry following NCX reversal has been implicated in a variety of pathophysiological conditions (Czyz and Kiedrowski, 2002; Floyd et al., 2005; Andrikopoulos et al., 2015; Gerkau et al., 2017; Brazhe et al., 2018; Secondo et al., 2020). In particular, the Ca^2+^ signalling mediated by reverse NCX has been shown to contribute to the astrocytic response to mechanical injury as well as to oligodendrocyte differentiation and myelin synthesis (Floyd et al., 2005; Boscia et al., 2012; Pappalardo et al., 2014; Hammann et al., 2018; Boscia et al., 2020). We also demonstrated in a previous study that the activation of the reverse mode of NCX1 induced by increased Na_V_-mediated currents played a fundamental role in Akt signalling and neuronal differentiation (Secondo et al., 2015). In the present study we show that the Ca^2+^ influx mediated by NCX3 working in the reverse mode significantly increased the amount of Ca^2+^ ions in the ER, a mechanism that has been demonstrated to be crucial for neuroprotection (Sirabella et al., 2009; Pannaccione et al., 2012; Sisalli et al., 2014).

In order to refill the ER Ca^2+^ store, a privileged pathway may occur between NCX working in the reverse mode and SERCA (Fameli et al., 2007; van Breemen et al., 2013). Furthermore, the tonic Ca^2+^ signal elicited by the application of thapsigargin in a calcium-free medium may suggest that SERCA is the main pump clearing cytosolic Ca^2+^ in hippocampal neurons. On the other hand, in Tg2576 neurons a significant alteration of the Ca^2+^ clearing mechanisms has been already reported (Lee et al., 2012). Among these mechanisms, the impairment of mitochondrial Ca^2+^ uptake, associated with increased mitochondrial reactive oxygen species and depolarization of mitochondrial membrane potential, may play an important role. Of note, the dysfunctional ER Ca^2+^ content was associated to the abnormal NCX3 reverse mode activity in Tg2576 neurons. In particular, our [Ca^2+^]_i_ measurements through Fura-2 AM fluorescence did not reveal any significant change in the cytosolic Ca^2+^ levels in the Tg2576 neurons compared with the WT neurons. In contrast, we observed a marked enhancement of the ER Ca^2+^ content in the transgenic neurons compared with the WT neurons, thus identifying the organellar Ca^2+^ dyshomeostasis as a putative biomarker of the AD pathology. Importantly, the inhibition of NCX3 through KB-R7943 significantly reduced the ER Ca^2+^ levels in the Tg2576 hippocampal neurons, hence showing that the increased Ca^2+^ influx through reverse NCX3 contributed to enhance the Ca^2+^ refilling into the ER of these neurons. On the other hand, the reduction of the ER Ca^2+^ content induced by KB-R7943 in the WT neurons indicated that NCX3 plays a key role in the ER Ca^2+^ replenishment in hippocampal neurons also in physiological conditions. These results are in line with several studies showing that NCX is located at the plasma membrane next to the junctional ER in numerous cell types (Blaustein et al., 2002; Lencesova et al., 2004; Fameli et al., 2007; Di Giuro et al., 2017), where it participates in ER Ca^2+^ handling. In agreement, many experimental data reported thus far have supported the concept that the entire NCX family is involved in the regulation of Ca^2+^ levels in the ER and in the sarcoplasmic reticulum (SR) by working in its reverse modality (Hirota et al., 2007; Lemos et al., 2007; Sirabella et al., 2009; Di Giuro et al., 2017). In particular, NCX has been recognized as an important mediator of Ca^2+^ influx in vascular smooth muscle cells (Poburko et al., 2007; Lee et al., 2012; Fameli et al., 2007; van Breemen et al., 2013), where its spatial and functional linkage to the SERCA pump allows the SR Ca^2+^ refilling that sustains [Ca^2+^]_i_ oscillations underlying smooth muscle contraction (Fameli et al., 2007; van Breemen et al., 2013). Moreover, it has been demonstrated that NCX is functionally coupled with the transient receptor channel protein 6 (TRPC6) at specialized SR/ER-plasma membrane junctions, where it mediates Ca^2+^ influx and hence regulates SR Ca^2+^ content following the localized intracellular Na^+^ concentration elevations mediated by TRPC6 (Poburko et al., 2007).

While in muscle cells the SR-mediated Ca^2+^ signalling is essential for excitation-contraction coupling, in central neurons the ER represents a dynamic Ca^2+^ reservoir indispensable for neuronal signalling. Moreover, as it constitutes the location of protein synthesis and post-translational folding, the ER may be also considered a regulator of cell fate. Indeed, any alteration of ER Ca^2+^ homeostasis, including severe changes in luminal Ca^2+^ levels, may trigger the unfolded protein response and ER stress, thus turning the ER in a potential source of cell death signals (Morishima et al., 2002; Verkhratsky and Petersen, 2002; Verkhratsky, 2004; 2005). Such a mechanism has been reported in a variety of AD models and is currently considered a crucial aspect of the AD pathogenesis (Verkhratsky and Toescu, 2003; Alberdi et al., 2013; Pannaccione et al., 2020; Salminen et al., 2020; Uddin et al., 2020). Indeed, Aβ oligomers may affect ER Ca^2+^ homeostasis by inducing an exaggerated Ca^2+^ release or interacting with ER-residing Ca^2+^ regulators such as ryanodine and inositol triphosphate receptors (Ferreiro et al., 2004; Costa et al., 2012; Alberdi et al., 2013; Wang and Zheng, 2019; Pannaccione et al., 2020). ER Ca^2+^ dyshomeostasis, in turn, may exert detrimental effects on neuronal function and survival and, likewise damaging, may favour the APP amyloidogenic processing and subsequent Aβ accumulation hence triggering a vicious circle (Paschen, 2001).

In this context, the NCX-mediated Ca^2+^ refilling into the ER, counteracting the reduction of the ER Ca^2+^ levels and preventing the ER stress cascade, may be determinant for neuronal survival. Of note, previous works have demonstrated that the ER Ca^2+^ refilling mediated by NCX, in particular the isoform 1, represents a protective mechanism helping cortical neurons to survive anoxic conditions (Sirabella et al., 2009; Sisalli et al., 2014). More importantly, our previous study on primary hippocampal neurons exposed to exogenous Aβ_1–42_ oligomers demonstrated that the increased reverse activity of NCX3 in the early phase contributed to a Ca^2+^ refilling into the ER, thus preventing an ER Ca^2+^ content reduction, ER stress activation and apoptotic cell death. Remarkably, the silencing or the knocking-out of the NCX3 gene prevented the enhancement of both the I_NCX_ and Ca^2+^ content in the ER stores and, in turn, activated caspase-12 (Pannaccione et al., 2012). Likewise, NCX3 loss in a late phase of Aβ exposure induced the activation of caspase-12 and the subsequent apoptotic cell death (Pannaccione et al., 2012). Interestingly, while NCX3 upregulation abruptly ceased in the late phase of a single exposure to exogenous Aβ_1–42_ oligomers, we did not observe any reduction of the I_NCX_ in the Tg2576 neurons over time in culture. This result could be explained by the fact that Tg2576 primary neurons progressively accumulate intracellular and extracellular Aβ over time in culture, with the highest Aβ burden observed at 19–21 DIV (Takahashi et al., 2004; Almeida et al., 2005). Importantly, an increased NCX activity had been already observed by Colvin and colleagues (1991) as Na^+^-dependent Ca^2+^ uptake in AD brains. In particular, an increased NCX activity was observed in the surviving neurons of AD brain areas suffering neurodegeneration. Although the exact mechanism involving the NCX upregulation was not clear, the authors concluded that NCX could have a role in the survival mechanisms implemented by the AD-affected neurons (Colvin et al., 1991). Based on our findings, we suggest that the neuroprotective effect of NCX observed in the AD neurons could be related to the ER Ca^2+^ remodelling.

We have previously shown that the I_Na_ carried by the Na_V_1.6 channels were upregulated in the Tg2576 hippocampal neurons (Ciccone et al., 2019). Interestingly, our co-expression studies revealed that in Tg2576 hippocampal neurons at 12 DIV, a time point displaying the maximum NCX3 activity and the upregulation of Na_V_1.6 currents (Ciccone et al., 2019), both the Na_V_1.6 and NCX3 immunoreactivities clustered along several cellular domains of both the soma and neurites, thus suggesting their possible functional coupling. In support of this observation, we provided evidence that the upregulation of the reverse I_NCX_ in the 12 DIV Tg2576 neurons was significantly reduced by inhibiting the Na_V_1.6 currents. In particular, we found that the widely used Na_V_ channel blocker TTX, by restricting the Na^+^ entry through the Na_V_1.6 channels, was able to significantly reduce the reverse I_NCX_ in the Tg2576 hippocampal neurons. Similarly, but to a lesser extent, also anisomycin, which induces the selective endocytosis of Na_V_1.6 and the subsequent reduction of Na_V_1.6 currents on the plasma membrane (Wittmack et al., 2005; Gasser et al., 2010; Ciccone et al., 2019), was able to decrease the I_NCX_ in the Tg2576 hippocampal neurons. These results suggested that the Na_V_1.6 over-expression and functional upregulation were responsible for the increased activation of the reverse mode of NCX in the Tg2576 hippocampal neurons. Of note, both TTX and anisomycin reduced the reverse I_NCX_ also in the WT neurons, a result suggesting that the Na^+^ influx through Na_V_1.6 channels could be one of the major Na^+^ sources inducing NCX3 reversal in hippocampal neurons also in physiological conditions. However, we cannot exclude the possibility that the modulation of the NCX3 expression pattern, namely the increase of the expression of the NCX3 truncated isoform, might contribute to enhance the reverse I_NCX_ in the Tg2576 hippocampal neurons to further potentiate the Ca^2+^ uptake.

The concept that the Na_V_ channels may play a key role in the interplay between the Na^+^ and Ca^2+^ cycling by modulating the NCX working modality has emerged from several studies. In particular, different experimental models of multiple sclerosis, astrogliosis and arrhythmogenesis have suggested that the Na_V_/NCX co-localization supports NCX reversal following Na_V_-mediated Na^+^ influx (Craner et al., 2004a; Craner et al., 2004b; Floyd et al., 2005; Larbig et al., 2010; Pappalardo et al., 2014; Radwański et al., 2016; Struckman et al., 2020). Indeed, although the rapid inactivation of Na_V_ currents could theoretically prevent a Na^+^ elevation sufficiently high to induce NCX reversal, the Na_V_/NCX proximity in a restricted microdomain may in fact generate a localized Na^+^ increase capable of activating the NCX reverse mode. The Ca^2+^ entry induced by NCX reversal following the Na_V_-mediated Na^+^ influx has been implicated in certain pathological conditions such as mechanical strain injury and *in vitro* astrogliosis (Floyd et al., 2005; Pappalardo et al., 2014). Pappalardo and colleagues (2014), in particular, showed that the [Ca^2+^]_i_ fluctuations through the reverse operation of NCX triggered by the Na_V_1.5 subunit contributed to the astrocytic response to mechanical injury (Pappalardo et al., 2014). Evidence suggesting that the Na_V_/NCX coupling might instead have a detrimental impact on cell functions has been provided by different studies focusing on axonal degeneration in multiple sclerosis. Craner and colleagues demonstrated that the Na_V_1.6 channels were extensively expressed on demyelinated axons and strongly associated with NCX in injured axonal regions (Craner et al., 2004a; Craner et al., 2004b). Based on these results, the authors speculated that Na_V_1.6 and NCX, inducing the accumulation of intra-axonal calcium, could participate in a cascade of deleterious events such as protease activation and mitochondrial failure leading to axonal injury. Nevertheless, due to the absence of functional analyses of the Na_V_1.6 and NCX activity, these studies did not clarify the exact implication of the increased Na^+^ influx nor whether the Ca^2+^ entering through reverse NCX underwent a further compartmentalization in order to trigger specific pathways. In this regard, it was shown that the inhibition of neuronal electrical activity with TTX reduced the number of myelinated fibers (Demerens et al., 1996) and decreased the proliferation of oligodendrocyte precursor cells (Barres and Raff, 1993), thus positively implicating TTX-sensitive Na^+^ currents and axonal electrical activity in the myelinogenesis process.

In the present study, we have found that the I_Na_ mediated by the Na_V_1.6 channel not only are crucial players in neuronal hyperexcitability (Patel et al., 2016; Wang et al., 2016; Ciccone et al., 2019; Zybura et al., 2021), but also modulate [Ca^2+^]_i_ by inducing the activation of the NCX reverse activity thus providing a Ca^2+^ source from the extracellular space to refill the ER Ca^2+^ stores. This evidence sheds new light on the Na_V_1.6 upregulation in AD neurons and suggests that its downstream effects may also depend on channel sub-cellular localization as well as on Na_V_1.6 interacting proteins.

## Conclusion

The present study has shown that NCX3 activity was upregulated in the reverse, Ca^2+^ influx mode in Tg2576 hippocampal neurons. Moreover, the enhanced reverse activity of NCX3 was associated with an increased Ca^2+^ refilling into the ER. Notably, functional experiments have indicated that the Na_V_1.6 channels, upregulated in the Tg2576 hippocampal neurons, were responsible for the increased activation of the NCX reverse mode, while confocal analyses have shown that their co-localization increased in the Tg2576 hippocampal neurons in comparison with the WT.

Collectively, these data reinforce the concept that the NCX3-mediated replenishment of the ER Ca^2+^ stores is a crucial mechanism intervening in neuronal homeostasis and promoting neuronal survival under pathological conditions such as those induced by Aβ_1–42_ oligomers. In addition, the observation that the reverse activity of NCX3 is driven by the Na^+^ influx mediated by Na_V_1.6 channels implies a possible functional link between Na_V_ channels and Ca^2+^ homeostasis and provides a new outcome of the Na_V_1.6 upregulation in AD hippocampal neurons.

## Data Availability

The original contributions presented in the study are included in the article/supplementary material, further inquiries can be directed to the corresponding author.
